# The Antineuroinflammatory Effect of Simvastatin on Lipopolysaccharide Activated Microglial Cells

**DOI:** 10.1155/2018/9691085

**Published:** 2018-11-07

**Authors:** Xinrui Zheng, Ye Liao, Jiu Wang, Shijie Hu, Gudepalya Renukaiah Rudramurthy, Mallappa Kumara Swamy, Komdur Channabasavaraju Rohit, Yangang Wang

**Affiliations:** ^1^Department of Neurosurgery, Xijing Hospital, Fourth Military Medical University, Xi'an, Shaanxi 710032, China; ^2^Department of Biotechnology, East West College of Science, Bengaluru 560091, India; ^3^Department of Crop Science, Faculty of Agriculture, Universiti Putra Malaysia, 43400 Serdang, Selangor, Malaysia; ^4^Department of Biotechnology, Sapthagiri College of Engineering, Bengaluru, India

## Abstract

Microglial cells, upon hyperactivation, produce proinflammatory cytokines and other oxidative stress mediators causing neuroinflammation, which is associated with the progress of many neurodegenerative diseases. Suppressing the microglial activation has hence been used as an approach for treating such diseases. In this study, the antineuroinflammatory effect of simvastatin was examined in lipopolysaccharide (LPS)-activated rat C6 glioma cells. The cell proliferation and cytotoxic effect of LPS and simvastatin on C6 glioma cells was evaluated by (MTT) assay. Neuroinflammation was induced in differentiated cell lines by treatment with 3.125 *μ*g/mL of LPS for 12 h. Upon induction, the cell lines were treated with different concentrations (3.125, 6.25, 12.5, 25, 50, 100 *μ*M) of simvastatin and incubated in a humidified CO_2_ incubator for 24 to 48 h. The optimum concentrations of LPS and simvastatin were found to be 3.125 *μ*g/mL and 25 *μ*M, respectively, with a cell viability of more than 90% at 24 h postincubation. Furthermore, proinflammatory marker expression was analyzed by flow cytometry and showed a decrease in interferon-*γ*, interleukin 6, nuclear factor-*κ*B p65, and tumor necrosis factor-*α* in simvastatin-treated and LPS-induced neuroinflammatory cells, and the mean fluorescent values were found to be 21.75 ± 0.76, 20.9 ± 1.90, 19.72 ± 1.29, and 16.82 ± 0.97, respectively, as compared to the untreated cells. Thus, we show that simvastatin has the potential to regulate the anti-inflammatory response in microglial cells upon LPS challenge. Hence, simvastatin can be employed as a potent anti-inflammatory drug against neuroinflammatory diseases and neurodegenerative disorders.

## 1. Introduction

The central nervous System (CNS) contains resident immune cells, such as the microglia and astrocytes, which are responsible for regulating the homeostasis of the brain [1]. The activation of resident immune cells in the CNS, termed as neuroinflammation, occurs due to immune-mediated disorders, viral infections, and neurodegenerative diseases, such as Alzheimer's disease (AD), multiple sclerosis (MS), Huntington's disease (HD), Parkinson's disease (PD), and amyotrophic lateral sclerosis (ALS) [2–4]. Microglial cells, which are one of the primary components of the brain's immune system, constantly survey the microenvironment in the CNS. They regulate the activities of the surrounding astrocytes and neurons during inflammation and mediate the process of tissue repair [4, 5]. Neuroinflammation induces microglial cells to produce several cytotoxic mediators, such as nitric oxide (NO), interleukin-1*β* (IL-1*β*), tumor necrosis factor *α* (TNF-*α*), reactive oxygen species (ROS), and prostaglandin E2 (PGE2). However, the overproduction of these mediators causes neurotoxicity, which might further lead to an amplification of the disease state, impaired repair mechanisms, and persistence of inflammatory stimuli [6–9]. Proinflammatory cytokines, such as interleukin 6 (IL-6), IL-1*β*, TNF-*α*, interferon gamma (IFN*γ*), nitric oxide synthase (NOS), and cyclooxygenase-2 (COX-2), play a significant role in modulating the inflammatory response by the microglial cells. The genes encoding these proinflammatory cytokines are, in turn, regulated by a set of molecules, such as nuclear transcription factor, kappa B (NF-*κ*B), and mitogen-activated protein kinase (MAPK) [8, 10, 11]. Hence, both NF-*κ*B and MAPK can act as potential targets for the development of various anti-inflammatory drugs [12].

Several earlier studies have reported the use of statins in the treatment of many diseases, including atherosclerosis and multiple sclerosis, and for improvement of cognition [13, 14]. Statins might reduce inflammation by disrupting the proinflammatory signaling pathways and also by modulating the functions of the endothelial cells [15, 16]. It is well established that anti-inflammatory effects of statins reduce the release of several inflammation mediators, including TNF-*α*, IL-1*β*, and NO [17]. However, not much is known about the neuroprotective functions of the anti-inflammatory actions of statins. The detailed investigation of this function could prove to be effective in developing drugs for preventing neuronal degeneration in diseases such as Parkinson's, Alzheimer's, and multiple sclerosis. Previous studies have shown the potential immune stimulatory effect of lipopolysaccharide (LPS), a bacterial endotoxin, and its potent applications in the activation of microglial cells and neuroinflammation [15, 18–21]. In the present study, neuroinflammation was induced in rat C6 glioma cell line using LPS. Further, the anti-inflammatory effect of simvastatin on LPS-activated microglial cells has been studied by the analysis of the expression of inflammation mediators.

## 2. Results

### 2.1. Viability Assay and Determination of Optimum Parameters

**Figure 1:**
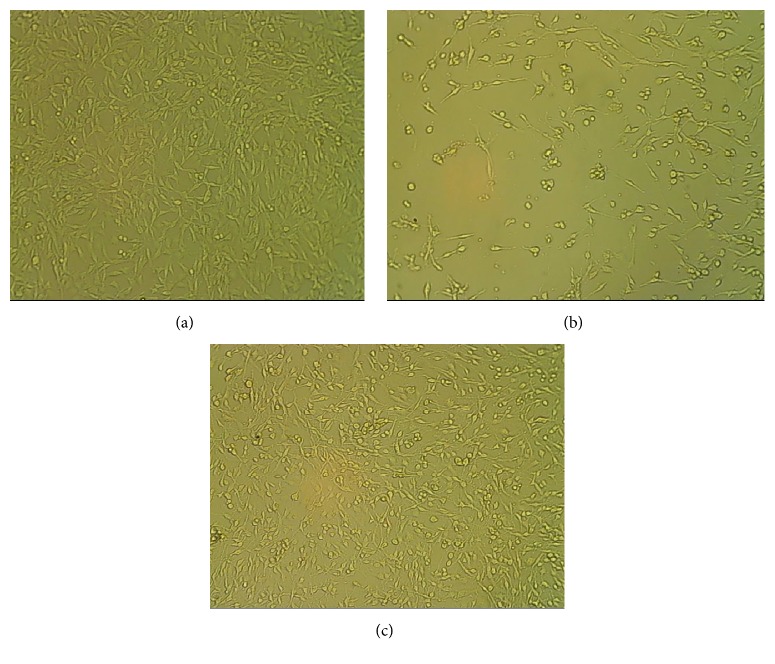
Comparative analysis of cytotoxic effect of drugs on C6 glioma cell line after 24 h of incubation (20x magnification). (a) Control (untreated) cell line, (b) treated with camptothecin, (c) treated with LPS (3.125 *μ*g/mL) + simvastatin (25 *μ*M).

**Figure 2:**
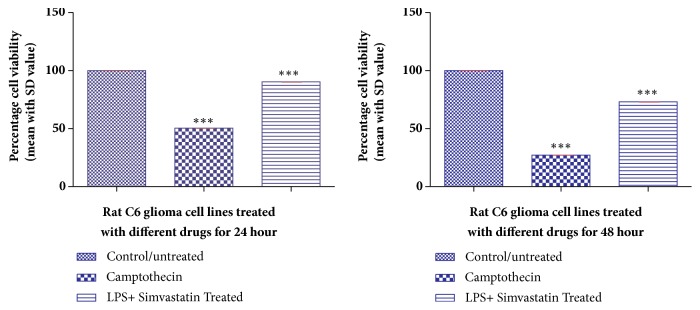
Histogram depicting the percentage cell viability of LPS (3.125 *μ*g/mL) + simvastatin (25 *μ*M) treated C6 glioma cell lines after 24 and 48 h of incubation. Data are reported as mean ± SD of 3 independent replicates. *∗∗∗* indicates* p* < 0.001 in comparison with control/untreated cells by one-way ANOVA followed by Dunnett's test for multiple comparison.

The cell viability upon treatment with LPS and simvastatin was tested using MTT [3-(4,5-Dimethyl-2-thiazolyl)-2,5-diphenyl-2H-tetrazolium bromide] assay with different concentrations of LPS, simvastatin, and a combination of both (Table 1) and revealed that the cell viability decreased with the increase in the concentration of LPS and simvastatin. About 99.89 ± 0.004% of cells were viable at LPS concentration of 3.125 *μ*g/mL, whereas simvastatin showed 92.17 ± 0.002% cell viability at 25 *μ*M after 24 h of incubation. However, the combined treatment with 3.125 *μ*g/mL of LPS and 25 *μ*M simvastatin resulted in cell viability of 90.17 ± 0.002%. The cell viability further decreased with an increase in incubation time (Table 1). DNA topoisomerase inhibitor camptothecin (15 *μ*M) was used as a positive control and restricted the cell viability to 50.45 ± 004% at 24 h of treatment and 27.14 ± 0.009% after 48 h of treatment. Hence, based on their lower cytotoxic effect, the optimum concentrations of LPS and simvastatin were chosen to be 3.125 *μ*g/mL and 25 *μ*M, respectively, with an incubation period of 24 h for proinflammatory studies. Further, comparision of control/untreated data with test data by Dunnett's test (*p *< 0.05) has shown high significance. However, the significance difference was not observed between the control cell lines and the cell lines treated with LPS at 3.125 *μ*g/mL, exhibiting cell viability of 99.89 ± 0.127 (*p* value >0.05); this indicates that LPS at 3.125 *μ*g/mL is not significantly cytotoxic to C6 glioma cell lines. The cytotoxic effect of the optimum concentrations of LPS and simvastatin on C6 cells after 24 h is shown in Figures 1 and 2.

### 2.2. Estimation of Proinflammatory Markers Expression

**Figure 3:**
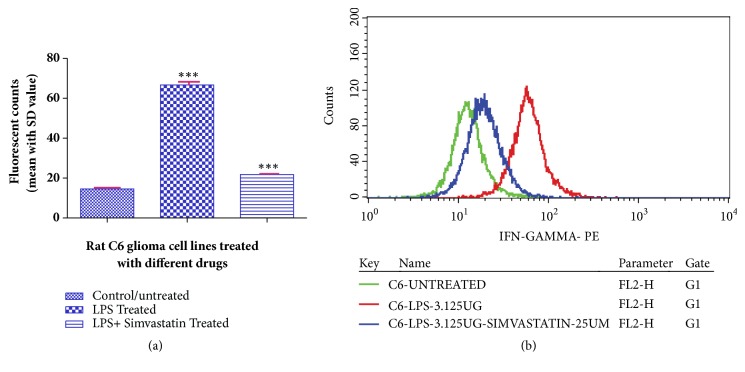
Histogram (a) and fluorescent overlay graph (b) representing the effect of simvastatin (25 *μ*M) on LPS (3.125 *μ*g/mL) induced rat glioblastoma cell line (C6) on the expression level of IFN-*γ*. Data are reported as mean ± SD of 3 independent replicates. *∗∗∗* indicates* p* < 0.001 in comparison with control/untreated cells by one-way ANOVA followed by Dunnett's test for multiple comparison.

The fluorometry assay and statistical analysis using the QuestPro software revealed the decrease of IFN-*γ* concentrations in simvastatin-treated LPS-induced cells. The mean fluorescent value was found to be 21.75 ± 0.76 with a classification variability (CV) of 48.43. However, LPS-induced cells showed a higher expression of IFN-*γ* having a mean fluorescent value of 66.69 ± 1.80 and CV value of 125.77. However, the control cells showed a lower mean value of 14.54 ± 0.70 (CV- 49.30), indicating the lower level of IFN-*γ* expression (Figure 3).

**Figure 4:**
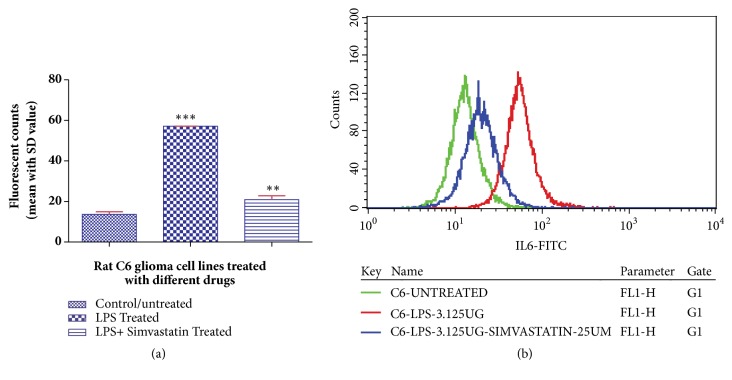
Histogram (a) and fluorescent overlay graph (b) representing the effect of simvastatin (25 *μ*M) on LPS (3.125 *μ*g/mL) induced rat glioblastoma cell line (C6) on the expression level of IL-6. Data are reported as mean ± SD of 3 independent replicates. *∗∗* indicates* p* < 0.01 and *∗∗∗* indicates* p* < 0.001 in comparison with control/untreated cells by one-way ANOVA followed by Dunnett's test for multiple comparison.

The estimation of IL-6 expression also revealed reduced levels of IL-6 in LPS- and simvastatin-treated cells with mean fluorescence of 20.9 ± 1.90 (CV- 45.15) as compared to that of only LPS-treated cells with mean fluorescence of 57.12 ± 0.31 (CV 43.56), whereas untreated C6 control cells showed a mean fluorescence of 13.63 ± 1.33 (CV- 40.88) (Figure 4).

**Figure 5:**
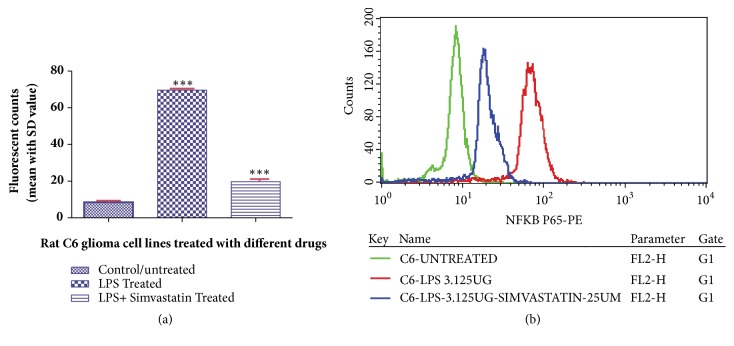
Histogram (a) and fluorescent overlay graph (b) representing the effect of simvastatin (25 *μ*M) on LPS (3.125 *μ*g/mL) induced rat glioblastoma cell line (C6) on the expression level of NF-*κ*B. Data are reported as mean ± SD of 3 independent replicates. *∗∗∗* indicates* p* < 0.001 in comparison with control/untreated cells by one-way ANOVA followed by Dunnett's test for multiple comparison.

**Figure 6:**
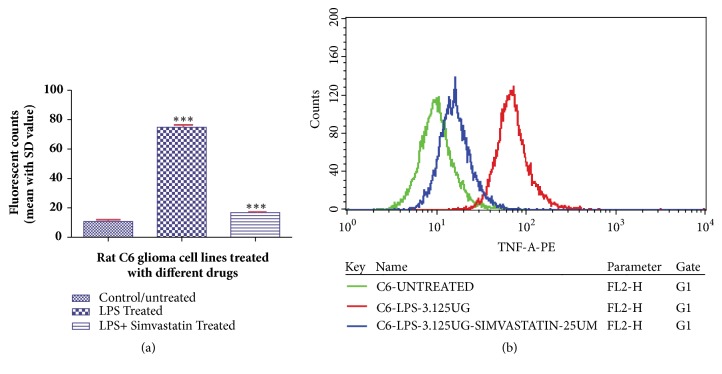
Histogram (a) and fluorescent overlay graph (b) representing the effect of simvastatin (25 *μ*M) on LPS (3.125 *μ*g/mL) induced rat glioblastoma cell line (C6) on the expression level of TNF-*α*. Data are reported as mean ± SD of 3 independent replicates. *∗∗* indicates* p* < 0.01 and *∗∗∗* indicates* p* < 0.001 in comparison with control/untreated cells by one-way ANOVA followed by Dunnett's test for multiple comparison.

Furthermore, the estimation of other proinflammatory markers such as NF-*κ*B and TNF-*α* in LPS- and simvastatin-treated cells also showed the decreased expression level as suggested by the statistical data. The mean fluorescent values of NF-*κ*B and TNF-*α* were found to be 19.72 ± 1.29 (CV- 34.65) and 16.82 ± 0.97 (CV- 45.11), respectively, in LPS- and simvastatin-treated cells (Figures 5 and 6). However, LPS alone treated and control cells showed higher mean fluorescent values in both NF-*κ*B and TNF-*α* markers. The mean fluorescent values in NF-*κ*B study were found to be 69.65 ± 1.05 (CV- 34.13) and 8.51 ± 1.01 (CV- 32.24) in LPS alone treated and control cells, respectively. Further, in case of TNF-*α* the mean values were found to be 74.85 ± 1.73 (CV- 135.15) and 10.71 ± 1.29 (CV-50.15), respectively, in LPS alone treated and control cells (Figures 5 and 6).

## 3. Discussion

Neuroinflammation is a complex process involving various cellular and biochemical reactions in the central nervous system stimulated due to brain injuries, infections, or neurodegenerative diseases, such as Parkinson's disease, Alzheimer's disease, and multiple sclerosis. Following the neuroinflammatory response, the activated glial cells release chemokines and cytokines, which act as inflammatory mediators and generate ROS and nitrogen species [22]. This allows damage recovery in the brain and promotes the restoration of the brain cells and their functions, which is crucial for alleviating neurodegenerative illnesses [23]. In neuroinflammatory diseases, inflammation is the main cause for the pathogenicity; hence, treatment with antineuroinflammatory agents/drugs can be potentially useful in controlling such diseases. In this regard, statins as a class of drugs have been reported to reduce the neuroinflammation; however, detailed mechanisms of action are yet to be identified and verified [24]. Several reports have demonstrated the proneuroinflammatory effects of LPS using different anti-inflammatory agents. However, the mechanisms involved in the inhibition of LPS-mediated neurotoxicity by simvastatin have not been completely elucidated. Therefore, we have validated the action of simvastatin in countering neuroinflammation in C6 glioma cells using flow cytometry assays, which are more advantageous for clinical monitoring and have not been extensively studied.

Statins are the globally used class of medication for the treatment of dyslipidemia and nonlipid related diseases, such as cardiovascular conditions including postmyocardial infarction [17, 25]. Several studies have shown the potential benefits of statins in the treatment and/or prevention of neurological disorders, such as Alzheimer's disease and multiple sclerosis, and this may be attributed to the anti-inflammatory action of statins [13, 14, 26]. The anti-inflammatory effect of simvastatin has also been established and it has been shown to contribute to the healing of injured hamster arteries [27]. Furthermore,* in vitro* and* in vivo* experimental studies using simvastatin have shown reduction in the expression of proinflammatory cytokines, such as MCP-1, IL-6, and IL-8, in patients with hypercholesterolemia [28]. Several other experimental studies have shown that pretreatment with simvastatin stimulates PPAR-*γ* receptors and inhibits NF-*κ*B expression, which in turn significantly reduces the proinflammatory cytokines [29]. In addition to the reduced expression of proinflammatory cytokines, statins also improve the balance between TNF-*α* and IL-10 in postmyocardial infarction in rats [30]. Simvastatin is also known to reduce proinflammatory cytokine expression through inhibition of TNF-*α*-mediated activation of NF-*κ*B [31, 32].

In the present study, inflammation was induced in C6 glioma cells with a nonlethal dose of LPS, followed by treatment with the drug, simvastatin, and the expression levels of proinflammatory cytokines were examined to assess the antineuroinflammatory effect of simvastatin. We show a significant reduction in the expression of proinflammatory cytokines, such as IFN-*γ*, IL-6, and TNF-*α* (Figures 1–4). As suggested earlier, IFN-*γ*, IL-6, and TNF-*α* are the critical proinflammatory elements produced in response to neuroinflammation [33–35], Thus, a significant reduction in the levels of these proinflammatory markers in this study suggests the antineuroinflammatory role of simvastatin in LPS-activated C6 glioma cells. Furthermore, statistical analysis revealed a lesser classification variability in both the tested cells (LPS- and simvastatin-treated) and control cells. The present study is in agreement with the previous studies, where simvastatin and rosuvastatin have been shown to inhibit TNF-*α* in microglia [17, 33, 36]. The proinflammatory cytokine, TNF-*α*, produced from the activated microglia, astrocytes, and neurons induces inflammatory responses through a cascade of events. Though astrocytes and neurons are also capable of producing TNF-*α*, microglia are the predominant contributors during neuroinflammation [34, 35]. It has been observed that simvastatin exerts a neuroprotective role in 6-hydroxydopamine challenged PC12 cells, partly by modulating N-methyl-D-aspartate receptors, matrix metalloproteinase-9, and TNF-*α* [37]. The present study shows a decrease in levels of IL-6 upon simvastatin treatment, suggesting its role in modulating the proinflammatory response by downregulating the expression of proinflammatory genes. However, the effect of simvastatin on the expression levels of IL-6 has been shown to give variable results with significant decrease in [28] and little [38] and/or no effect of simvastatin on IL-6 levels [39, 40]. NF-*κ*B is a key regulator of the transcription of several genes, including proinflammatory enzymes, proinflammatory transcription factors, chemokines, cytokines, adhesion molecules, and several other factors, and is the central regulator of inflammation [11]. Activated microglial NF-*κ*B is closely associated with the release of ROS and proinflammatory cytokines, including IFN-*γ*, TNF-*α*, and IL-1*β*, which can cause secondary neurotoxicity and inflammation-induced neurodegeneration [11, 41, 42]. Likewise, studies have confirmed that LPS induces neuroinflammation-associated gene expression via the NF-*κ*B pathway. Hence, drugs inhibiting NF-*κ*B pathway signaling could prevent LPS-induced neuroinflammatory processes [8, 43, 44]. Plant hormone osmotin has been shown to alleviate LPS-induced neuroinflammation and memory impairment via regulation of the TLR4/NF-*κ*B signaling pathway [44]. On the similar lines, we suggest that simvastatin can attenuate LPS-induced NF-*κ*B signaling. Hence, our data corroborates the previous findings that decreased NF-*κ*B expression is effective in reducing the risk of secondary neurotoxicity and inflammation-induced neurodegeneration. In addition, our results show a reduction in the levels of proinflammatory cytokines, such as IFN-*γ*, IL-6, and TNF-*α* in simvastatin-treated C6 glioma cell line. Our study confirms that simvastatin can regulate neuroinflammation by negatively modulating the NF-*κ*B pathway and decreasing the expression of other proinflammatory mediators, mainly cytokines. Thus, simvastatin could be evaluated further in order to characterize its potential as an anti-inflammatory drug against neuroinflammation and related diseases.

## 4. Materials and Methods

### 4.1. Chemicals and Reagents

LPS from* Escherichia coli* O111:B4 (Cat no: L2630), simvastatin (Cat no: S6196), Dimethyl sulfoxide (DMSO), and Dulbecco's Phosphate Buffered Saline (D-PBS; Cat no. TL1006) were procured from Sigma-Aldrich, St. Louis, Missouri, USA. DMEM (Cat no. AL111), Fetal Bovine Serum (FBS; Cat no. RM10432), and MTT reagent (5 mg/ml) (Cat no. 4060) were procured from Himedia Laboratories Pvt. Ltd., Bengaluru, India. NF-*κ*B p65-PE (Cat no: 558423), TNF-*α*–PE (Cat no. 559321), IFN-*γ*-PE (Cat no. 562016), and IL6-FITC (Cat no. 554544) were obtained from BD India Pvt Ltd., Bengaluru, India.

### 4.2. Cell Lines and Proliferation

Rat glioblastoma cell line (C6) was obtained from the National Centre for Cell Sciences (NCCS), Pune, India. C6 glioma cells are considered as a common* in vitro* model system, as these cells retain several characteristics of glioblastoma multiforme (GBM) [45, 46]. The cell lines were revived and proliferated in Dulbecco's minimum essential medium (DMEM) at 37°C in a humidified CO_2_ incubator (5%) (Heal Force Bio-Meditech Holdings Ltd., Shanghai, China). The cell lines were preserved in liquid nitrogen until further use.

### 4.3. Viability Study by MTT Assay

Viability study was carried to determine the optimum concentration of LPS and simvastatin used in the present study. The MTT assay was carried out by the standard drug camptothecin at a concentration of 15 *μ*M as a positive control and untreated cells as control cell lines. The LPS stock was prepared in double distilled water, while the camptothecin and simvastatin stocks were prepared in 100% Dimethyl sulphoxide (DMSO), respectively. For the viability study, 200 *μ*L C6 cell line suspension was seeded in 96-well plate at the cell density of 20,000 cells per well. The cells were proliferated for about 12 h in DMEM containing 10% fetal bovine serum (FBS) in a humidified CO_2_ (5%) incubator. After 12 h of the growth, the cell lines were treated separately with a different concentration of LPS (3.125, 6.25, 12.5, 25, 50, and 100 *μ*g/mL) and simvastatin drug (6.25, 12.5, 25, 50, and 100 *μ*M) and incubated for different time period, i.e., 24 and 48 h at 37°C in a humidified CO_2_ (5%) incubator. The positive and negative controls were also run simultaneously. After the incubation period, the spent media were removed and 0.5 mg/mL of MTT reagent (Himedia Laboratories Pvt. Ltd., Bengaluru, India) was added and incubated for 2 h at 37°C. After the completion of incubation time, the MTT reagent was removed and the Formazen crystals formed in the wells were dissolved with 100 *μ*L of DMSO and the optical density (OD) was read in a ELx808 absorbance microplate reader (BioTek Instruments, Inc., Mumbai, India) at 570 nm. The OD values in each concentration were measured and the viability of the cells was determined by using the formula

(1) The  percentage  of  viability=OD  of  the  test  compoundOD  of  the  untreated  cells×100.

### 4.4. Induction of Neuroinflammation and Simvastatin Treatment

LPS from* Escherichia coli* O111:B4 (Sigma-Aldrich, St. Louis, Missouri, United States) has been used in the neuroinflammation of C6 gliomacell lines. Simvastatin used in the present study was procured from Sigma-Aldrich, St. Louis, Missouri, United States. The differentiated cell line was induced for neuroinflammation by treating with 3.125 *μ*g/mL of LPS for 12 h. Upon induction, the cell lines were treated with different concentrations (6. 25, 12.5, 25, 50, 100 *μ*M) of simvastatin and incubated for 24 h and 48 h to determine the optimum concentration of simvastatin and incubation time. Finally, the MTT assay was performed to determine the percentage of cell viability in each concentration of simvastatin as mentioned in Section 4.2.

### 4.5. Determination of Proinflammatory Markers Expression

To determine the proinflammatory markers expression, the C6 glioma cells (1 × 105 cells/well) were seeded in DMEM in a 6-well plate and allowed to proliferate for 12 h in a humidified CO_2_ incubator. After 12 h, of incubation the cells were then cultured in the DMEM containing 3.125 *μ*g/mL of LPS for 24 h. Following the incubation, cells were washed twice with phosphate buffered saline (PBS) and transferred into the DMEM containing simvastatin (25 *μ*M) and incubated for 24 h in a humidified CO_2_ incubator. Simultaneously, the cell lines treated with LPS alone and cells without any treatment were used as positive and negative control, respectively. After the completion of incubation period, the cells were trypsinized and harvested by centrifugation at 214 g for 5 min. The cells were then fixed in 70% ice cold ethanol by incubating at -20°C for 30 min, washed twice with PBS, and used in the estimation of different proinflammatory markers.

For the estimation of proinflammatory markers such as NF-*κ*B P65, TNF-*α*, IFN-*γ*, and IL6; the test cell line (simvastatin-treated cells after the LPS-induced inflammation), positive control and negative control were treated with 20 *μ*L of fluorescein isothiocyanate (FITC)-conjugated antibodies against NF-*κ*B P65, TNF-*α*-, IFN-*γ*, and IL6 antibodies (BD India Pvt Ltd., Bengaluru, India) for 60 min at room temperature in the dark. Upon completing the incubation, the cells were washed with 1 mL of phosphate buffered saline (PBS) and suspended in 0.5 mL of PBS. The cells were then analyzed for relative fluorescence intensity using BD FACS Callibur™ (BD India Pvt Ltd., Bengaluru, India). The relative fluorescence intensity values were analyzed by the cell QuestPro software (Quest Software, California, United States).

### 4.6. Statistical Analysis

All tests were repeated three times and the results are expressed as the mean ± standard deviation (SD). One-way analysis of variance (ANOVA) was carried out to compare the data. QuestPro software (Quest Software, California, United States) was used to analyze all flow cytometry parameters. Further, multiple comparisons were done through Dunnett's test by comparing the test data with control/untreated data to determine the statistically significant differences. The Dunnett's test was performed at* p* < 0.05 level using GraphPad Prism (version 5.0) statistical software.

## 5. Conclusion

The present study revealed that simvastatin exerts an anti-inflammatory effect on LPS-induced neuroinflammation in the C6 glioma cell line by reducing the expression levels of many proinflammatory cytokines. The anti-inflammatory properties of simvastatin in microglial cells challenged with LPS suggested its possible use as a potent anti-inflammatory drug against neuroinflammation in activated microglia. Thus, simvastatin may be helpful in fighting several neurodegenerative disorders. However, further research is needed to elucidate the detailed molecular mechanisms of action of simvastatin.

## Figures and Tables

**Table 1:** Cytotoxic effect of LPS, simvastatin, and LPS+ simvastatin on rat C6 glioma cell lines at different concentrations and incubation times. Data is expressed as the mean ± SD (each treatment, n = 3). ^ns^  Nonsignificant. *∗∗∗* indicates *p* < 0.001 in comparison with control/untreated cells by one-way ANOVA followed by Dunnett's test for multiple comparison.

**Drug (Test compound)**	**Concentration**	**Cell viability (**%**)**	
Incubation Time		**24 h**	**48 h**

Camptothecin (Positive control)	15 *µ*M	50.45 ± 0.095^*∗∗∗*^	27.14 ± 0.109^*∗∗∗*^

Control/untreated cell line (Negative control)	-	100 ± 0.005	100 ± 0.005

LPS	3.125 *µ*g/mL	99.89 ± 0.127^ns^	95.20 ± 0.092^*∗∗∗*^
	6.25 *µ*g/mL	96.90 ± 0.119^*∗∗∗*^	89.10 ± 0.076^*∗∗∗*^
	12.5 *µ*g/mL	94.60 ± 0.085^*∗∗∗*^	85.20 ± 0.088^*∗∗∗*^
	25 *µ*g/mL	90.10 ± 0.100^*∗∗∗*^	77.50 ± 0.067^*∗∗∗*^
	50 *µ*g/mL	87.30 ± 0.066^*∗∗∗*^	72.80 ± 0.089^*∗∗∗*^
	100 *µ*g/mL	85.50 ± 0.097^*∗∗∗*^	69.50 ± 0.046^*∗∗∗*^

Simvastatin	6.25 *µ*M	98.10 ± 0.109^*∗∗∗*^	85.80 ± 0.097^*∗∗∗*^
	12.5 *µ*M	96.30 ± 0.098^*∗∗∗*^	83.00 ± 0.086^*∗∗∗*^
	25 *µ*M	92.17 ± 0.078^*∗∗∗*^	79.30 ± 0.101^*∗∗∗*^
	50 *µ*M	90.07 ± 0.102^*∗∗∗*^	75.20 ± 0.098^*∗∗∗*^
	100 *µ*M	86.90 ± 0.097^*∗∗∗*^	70.80 ± 0.095^*∗∗∗*^

LPS (3.125 *µ*g/mL) + Simvastatin at different concentrations	6.25 *µ*M	98.00 ± 0.096^*∗∗∗*^	81.25 ± 0.083^*∗∗∗*^
	12.5 *µ*M	94.80 ± 0.078^*∗∗∗*^	78.75 ± 0.066^*∗∗∗*^
	25 *µ*M	90.27 ± 0.099^*∗∗∗*^	73.00 ± 0.047^*∗∗∗*^
	50 *µ*M	87.00 ± 0.104^*∗∗∗*^	66.70 ± 0.072^*∗∗∗*^
	100 *µ*M	84.90 ± 0.112^*∗∗∗*^	64.80 ± 0.109^*∗∗∗*^

## Data Availability

The data used to support the findings of this study are available from the corresponding author upon request.
